# Levels of heavy metal cadmium in rice (*Oryza sativa* L.) produced in Taiwan and probabilistic risk assessment for the Taiwanese population

**DOI:** 10.1007/s11356-020-11902-w

**Published:** 2021-02-04

**Authors:** Keng-Wen Lien, Min-Hsiung Pan, Min-Pei Ling

**Affiliations:** 1 Institute of Food Science and Technology, National Taiwan University, No. 1, Section 4, Roosevelt Rd, Da’an District, Taipei City, 10617 Taiwan; 2 Department of Food Science, National Taiwan Ocean University, No. 2, Beining Rd., Jhongjheng District, Keelung City, 202 Taiwan; 3 Department of Medical Research, China Medical University Hospital, China Medical University, No. 91, Xueshi Rd, North District, Taichung City, 404 Taiwan; 4 Department of Health and Nutrition Biotechnology, Asia University, No. 500, Liufeng Rd, Wufeng District, Taichung City, 41354 Taiwan

**Keywords:** Cadmium, Hazard index, Lifetime average daily dose, Monte Carlo simulation, Probabilistic risk assessment, Taiwan rice

## Abstract

Cadmium (Cd) is a toxic pollutant that is widely spread through industrial production and agricultural practices. Epidemiological data has revealed that lifetime exposure to environmentally relevant levels of Cd increases the risk of developing various organ diseases, including chronic kidney, heart, and lung diseases, as well as nervous tissue disorders. This study assessed Cd levels in rice to determine the health risks associated with rice consumption in various age-gender subgroups in Taiwan. The distribution of Cd concentration, the lifetime average daily dose (LADD), and the hazard index (HI) were estimated by Monte Carlo simulation. In the general population, the 50th percentile LADD of Cd for male rice consumers between the ages of 19–65 years was 0.06 μg/kg body weight per day, and the hazard index (HI) 50th, 90th, and 95th percentiles were 0.16, 0.69, and 1.54, respectively. According to the HI heat map for the exposure of the general population to Cd from rice in Taiwan, the highest exposure to Cd was noted in the Yilan area (HI 0.64). Therefore, rice production in the Yilan area should be further monitored to evaluate the levels of Cd contamination.

## Introduction

Rice is an important source of energy, vitamins, and minerals, as well as a moderate source of easily digestible proteins and carbohydrates (Food and Agriculture Organization of the United Nations [FAO] 2003; Fresco 2005; Heinemann et al. 2005; Pastorelli et al. 2018). Rice forms a major part of the Taiwanese diet, and 90% of the rice consumed is domestically produced. Rice consumption in Taiwan has been estimated to be 1.43 million tonnes per annum (Council of Agriculture of Taiwan [COA] 2019). However, due to a disordered arrangement of land use, factories have been built near farmlands. Irrigation water has been chronically polluted by industrial wastewater, which is the major cause of farmland pollution. Heavy metals and toxic organic matter are continually discovered within farmland soil and have caused serious damage to the agricultural production of Taiwan (Environmental Protection Agency [EPA] 2019).

Rice was first found to be contaminated with cadmium (Cd) in 1982; the contaminated rice was later referred to as “Cd rice” from paddy fields in Taoyuan County, Republic of China (Taiwan), and subsequent discovery of rice contaminated with Cd was reported in many areas in Taiwan (EPA 2019). The irrigation water used in these rice paddies was contaminated by wastewater with high concentrations of Cd from nearby chemical factories producing polyvinyl chloride stabilizers. Compared with other crops, rice has a greater ability to absorb toxic elements from soil and water. Paddy plants are grown under flooded conditions, which enable toxic elements to be absorbed by the roots of the plants and accumulate (Huang et al. 2016). The anaerobic conditions in paddy soil increase the bioaccessibility of toxic elements for rice. Since toxic elements accumulate, are non-biodegradable, and have long biological half-lives, their presence in marketed rice is essentially unavoidable (Morekian et al. 2013; Sugita 1978; Jaishankar et al. 2014). Heavy metal contamination becomes a concern as the metals become biomagnified and ultimately pose a potential threat to human health (Khan et al. 2016; Thakur et al. 2016).

The principal factor determining how much Cd is absorbed is the route of exposure. Generally, ingestion is the major route of exposure of Cd; most orally ingested Cd passes through the gastrointestinal tract unchanged as normal individuals absorb only about 6% of ingested Cd (Agency for Toxic Substances and Disease Registry [ATSDR] 1999). After exposure to normal dietary concentrations of Cd (10–30 μg/day), about 50% of the body burden is found in the kidneys, about 15% in the liver, and about 20% in the muscle (Nordberg and Kjellstrom 1979). The biologic half-life of Cd in the kidney and liver is estimated to be between 6 and 38 and between 4 and 19 years, respectively (ATSDR 2006). The human body does not have effective pathways for Cd elimination. Absorbed Cd is eliminated from the body primarily in urine. The rate of excretion is low, probably because Cd remains tightly bound to metallothionein, which is almost completely reabsorbed in the renal tubules. Due to slow excretion, Cd accumulation in the body can be significant (ATSDR 2006).

Cd is a non-essential heavy metal that has been widely implicated in toxicity and carcinogenicity. It is a non-redox metal and therefore unable to participate in reactions itself but causes oxidative stress by generating reactive oxygen species (ROS) (Garnier et al. 2006). ROS react with lipids, proteins, nucleic acids, and pigments causing lipid peroxidation, membrane damage, and inactivation of enzymes, which result in toxic effects. Long-term exposure even to low levels of Cd causes diseases, including kidney dysfunction. Additionally, Cd is a possible risk factor for osteoporosis even at low levels of environmental exposure (Åkesson et al. 2006; Gallagher et al. 2008). Some studies have found that exposure to Cd may increase the incidence of renal, prostate, and breast cancer in the general population; however, further studies are warranted to investigate these correlations (Verougstraete et al. 2003; Ilyasova and Schwartz 2005). Cho et al. (2013) reported a positive association between the dietary intake of Cd and risk of cancer, particularly with hormone-related cancers, in studies conducted in Western countries. Another study revealed that daily intakes of Cd in children and of mercury in adults exceeded the safe limits (Huang et al. 2013). Since 2010, the Taiwanese government has been performing a Cd rice survey for the protection of public health.

Considering the high levels of consumption of rice in Taiwan, the objectives of this study are to (1) measure the levels of Cd in domestically produced rice in Taiwan and (2) assess the potential non-carcinogenic health risks linked to the intake of metals from rice consumption using the hazard index (HI) for the total population and consumers, including children, teenagers, adults, and older adults.

## Materials and methods

### Samples

**Fig. 1:**
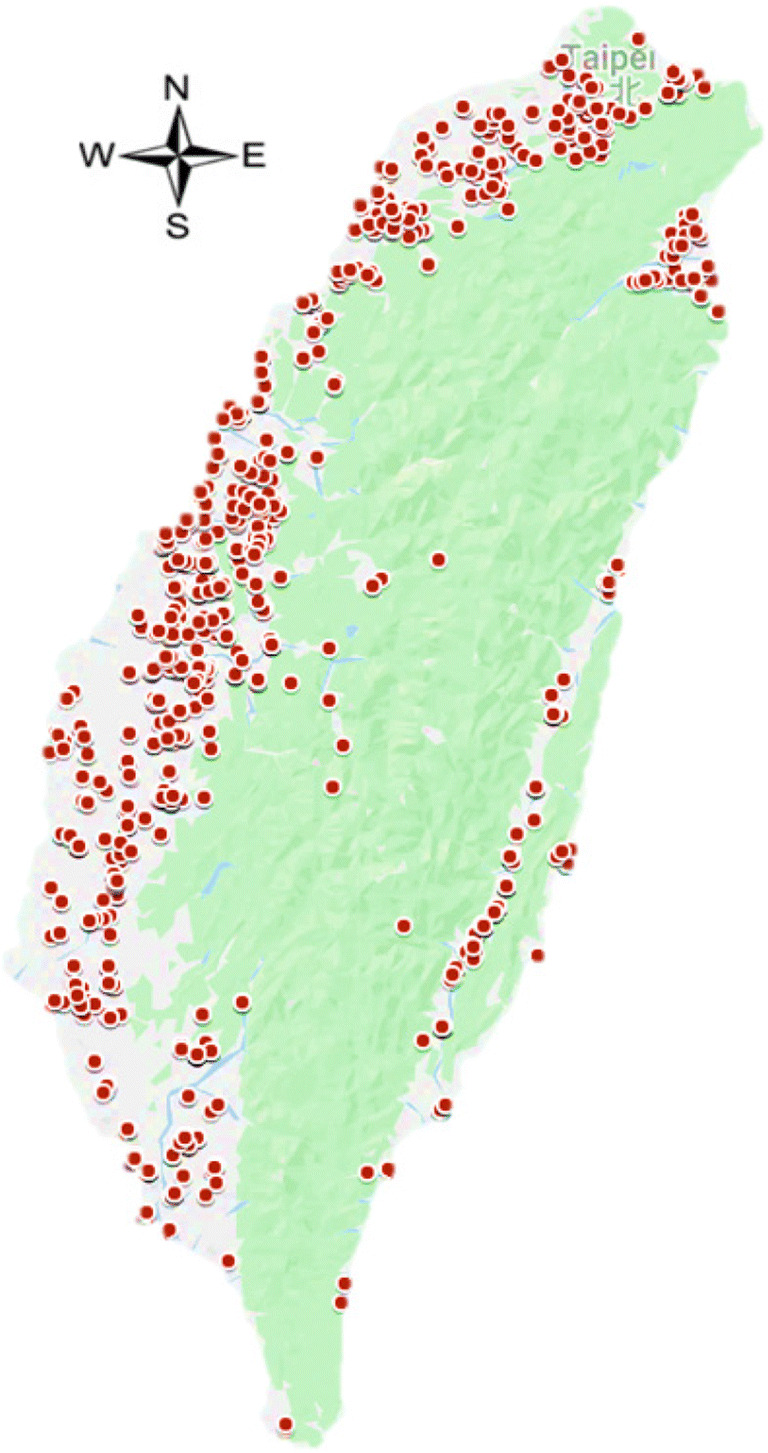
Location of sampling sites in Taiwan

A total of 1581 domestic rice samples were collected from local farmers’ associations in production areas or randomly collected from local markets. The samples were sealed in plastic bags, at least 600 g per sample. The areas of origin for the rice samples were as follows: Changhua, Chiayi, Hsinchu, Hualien, Kaohsiung, Keelung, Miaoli, Nantou, New Taipei, Penghu, Taichung, Tainan, Taipei, Taitung, Taoyuan, Yilan, and Yunlin. The sampling sites of rice in Taiwan are shown in Fig. 1. Rice samples were ground using an agate mortar, sealed in a plastic box, and stored at 4 °C until analysis.

### Heavy metal Cd analysis

The analysis of Cd in the rice samples was conducted according to the guidelines released by the Taiwan Food and Drug Administration (TFDA). This method is applicable to the determination of Cd in rice when coupled an inductively with plasma mass spectrometry (ICP-MS 7700, Agilent Technologies, USA) analysis (TFDA 2014).

For the internal standard solution, 10 mL of rhodium internal standard was added to a 100-mL volumetric flask along with 90 mL of 1% nitric acid and transferred to a digestion storage tube.

For the standard solutions, 0.1 mL of each Cd reference standard was added to a 50-mL volumetric flask along with 49.9 mL of 1% nitric acid and transferred to digestion storage tubes. For use, the appropriate amounts of standard stock solutions and the internal standard solution were mixed, diluted to 1–25 ng/mL with 1% nitric acid (containing 10 ng/mL internal standard), and transferred to digestion storage tubes.

To prepare sample solutions, 300 g of the edible parts of each rice sample was blended. Approximately 1 g of the blended sample was transferred to a digestion storage tube. Subsequently, 0.2 mL of the internal standard solution and 10 mL of nitric acid (trace metal grade) were added, and the samples were digested using a graphite block digester. Digestion was performed at 60 °C for 30 min, and the temperature was subsequently increased to 95 °C for 90 min. After cooling to room temperature, each sample was diluted to 20 mL with deionized water and filtered with a membrane filter. The filtrate was used as the sample solution. Subsequently, 0.2 mL of the internal standard solution and 10 mL of nitric acid (trace metal grade) were added to a blank digestion storage tube, and the same procedure described above for the blank solution was performed.

The standard solutions were injected into the ICP-MS at the appropriate rate to prepare the standard curve, and the analysis was performed according to the following conditions. The standard curve of Cd was established using the ratios of the signal intensity of Cd to that of rhodium versus the added concentrations.

The ICP-MS was operated under the following conditions: the radiofrequency power of plasma was 1300 W; the flow rate of argon plasma was 15 L per min; the flow rate of auxiliary argon was 0.2 L per min; the flow rate of atomizing argon was 0.8 L per min; and the atomic mass of Cd was 114, 112, or 111.

For quantification, the sample solution, blank solution, and the standard solutions were injected into the ICP-MS separately at the appropriate rate, and the analysis was performed according to the conditions listed above. The limit of quantification (LOQ) was 0.2 mg/kg. All chemicals used were of analytical reagent grade. The referral laboratory has been certified for the chemical analyses of foods under the International Organization for Standardization 17025 guidelines.

### Hazard identification for Cd

According to the United States EPA (1985), the toxicokinetic model predicts that the no-observed-adverse effect level for chronic exposure to Cd is 0.005 g Cd/kg per day from water and 0.01 mg Cd/kg per day from food. Thus, a reference dose (RfD) of 0.0005 mg Cd/kg per day (water) was used in our calculations. This was based on an estimated no-observed-adverse effect level of 0.005 mg Cd/kg per day for Cd in drinking water and an uncertainty factor of 10. The equivalent RfD for Cd in food is 0.001 mg Cd/kg per day. Cd has been classified by the International Agency for Research on Cancer (IRAC) as a group 1 human carcinogen (IARC 1993; IARC 2012). A provisional tolerable weekly intake of 7 μg/kg bw for Cd (FAO/WHO 2005) and provisional tolerable monthly intake of 25 μg/kg bw were established by the Joint Food and Agriculture Organization of the United Nations/World Health Organization expert committee on food additives (JECFA 2010, 2013). This provisional intake was reevaluated by the European Food Safety Authority (EFSA) to remain below the modified reference point of 1 μg Cd per gram creatinine in urine. The EFSA calculated that the average daily dietary intake of Cd should not exceed 0.36 μg/kg bw per day, which was set a lower tolerable weekly intake of 2.5 μg/kg bw (EFSA 2009, 2011).

### Exposure assessment

Taiwanese rice consumption data were derived from the National Food Consumption Database (Nutrition and Health Survey in Taiwan [NAHSIT] 2018). We calculated the dietary exposure to Cd from rice according to age through the following formula: LADD = (C × IR × AF)/BW × ED/AT, where *C* is the Cd concentration (mg/kg) in rice, IR is the daily consumption of rice (g/day) for each age group according to the NAHSIT survey results for the diet of the Taiwanese population, AF is the absorption factor (%) (100% in the present study), BW is the body weight (kg) based on the average body weight of Taiwanese individuals in each age group, ED is the duration of exposure (year), and AT is the average time (year). Samples with a Cd concentration above the limit of quantification were considered positive. When calculating the mean levels and denoting all values less than the limit of detection by zero, the mean concentration of Cd accounts for all individual values. The HI was calculated as follows: HI = LADD/RfD, where the *RfD* is the benchmark dose lower limit value of 0.36 μg/kg bw per day set by the EFSA in 2011.

### Data analysis

The data were analyzed using the SPSS version 10.0 software (SPSS Inc., Chicago, IL, USA.). The mean, minimum, and maximum values and standard deviations were calculated to describe the Cd content in the rice samples. We used the Crystal Ball software (Crystal Ball 2000 Professional Edition, Version 5.2.2, Decisioneering Inc., Denver, Colorado, USA) for Microsoft Excel 2003 to conduct Monte Carlo simulations. Input parameters were Cd concentration, daily food consumption rate, and consumer body weight. Each parameter had assigned minimum and maximum values, and arbitrary probability density distributions were obtained for individual parameters. The exposure rate to Cd was repeatedly calculated with the parameters by random sampling in accordance with the assigned probability density distribution. The number of iterative calculations was set to 100,000.

## Results and discussion

### Cd concentrations in rice

**Fig. 2:**
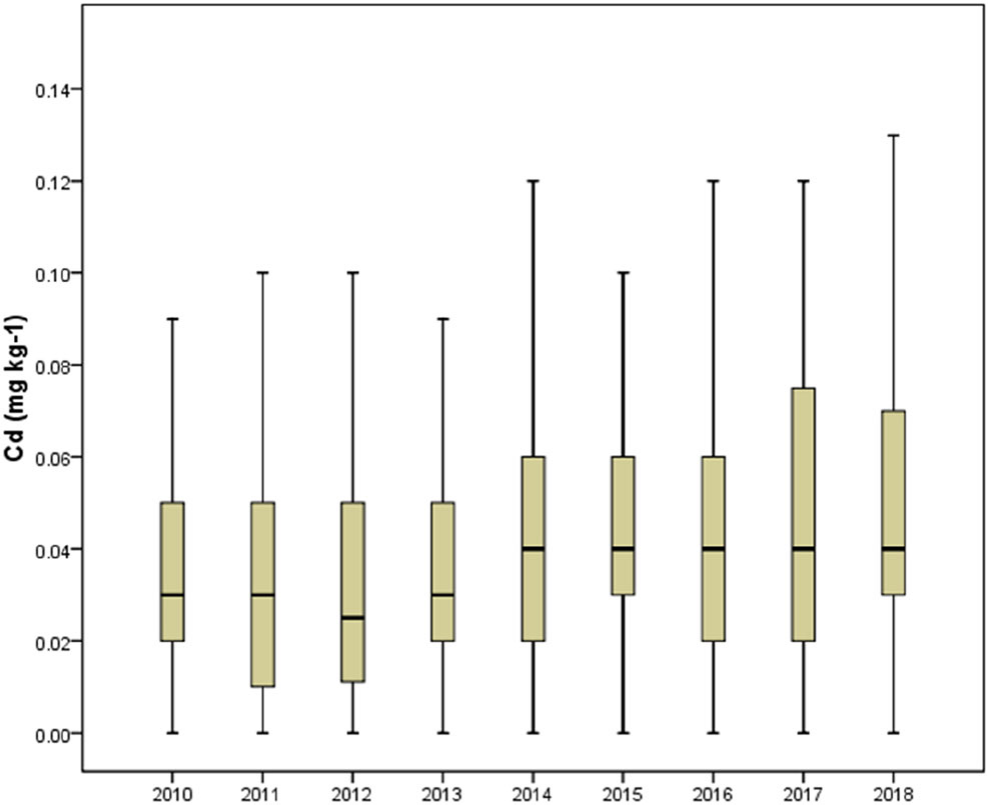
Box plot for the levels of cadmium in rice from 2010 to 2018 (Data from bottom to top were minimum, first quartile, median, third quartile, and maximum.)

Cd was detected in 89% of the rice samples. The average Cd level in rice was 0.04 ± 0.04 mg/kg (*n* = 1581). The mean Cd concentrations in rice obtained from different areas are summarized in Table 1. All Cd concentrations in the rice samples were below the maximum level (0.4 mg/kg) permitted by Taiwan Food Regulations (TFDA 2018). The box plot showing minimum, first quartile, median, third quartile, and maximum for the levels of Cd in rice from 2010 to 2018 is shown in Fig. 2. When comparing the changes over time, we found that the average Cd concentrations in rice slightly increased from 0.04 mg/kg in 2010 to 0.06 mg/kg in 2018. Lin et al. (2004) surveyed the heavy metal content of Taiwanese rice between 1990 and 1991. Their results revealed an average of Cd content in the rice samples of 0.01 mg/kg (ranging from not detected [N.D.] to 0.20 mg/kg) (Lin et al. 2004). Chiu et al. (2005) found that the average content of Cd in Taiwan’s first (June) and second (November) cultivations of rice crop was 0.04 mg/kg (N.D. to 0.12 mg/kg) and 0.05 mg/kg (0.01–0.24 mg/kg), respectively. Although studies of Lin et al. (2004) and Chiu et al. (2005) were performed several years ago, they reported similar or slightly lower levels of Cd compared with our study.

**Table 1:** Concentration of cadmium (mg/kg fresh weight) in Taiwanese commercial rice from 2010 to 2018

Area	Number	Cadmium concentration			
		Mean	SD	Median	Max
Changhua	210	0.03	0.03	0.03	0.16
Chiayi	138	0.03	0.02	0.02	0.12
Hsinchu	102	0.06	0.04	0.04	0.17
Hualien	115	0.03	0.02	0.03	0.10
Kaohsiung	96	0.03	0.03	0.02	0.14
Keelung	39	0.05	0.04	0.04	0.24
Miaoli	76	0.05	0.02	0.04	0.12
Nantou	32	0.07	0.03	0.04	0.13
New Taipei	40	0.04	0.04	0.04	0.15
Pingtung	73	0.03	0.02	0.05	0.13
Taichung	120	0.07	0.03	0.04	0.22
Tainan	90	0.03	0.04	0.02	0.25
Taipei	40	0.05	0.08	0.04	0.37
Taitung	75	0.02	0.02	0.03	0.07
Taoyuan	95	0.08	0.06	0.05	0.39
Yilan	69	0.10	0.08	0.03	0.27
Yunlin	171	0.04	0.03	0.04	0.22
Total	1581	0.04	0.04	0.03	0.39

*SD* standard deviation, *Max* maximum

Studies of rice in other countries have shown Cd levels ranging between N.D. and 0.07 mg/kg (Alam et al. 2002; Gorbunov et al. 2003; Jorhem et al. 2008; Phuong et al. 1999; Shimbo et al. 2001; Wolnik et al. 1985). In Japan, rice is a major source of Cd intake for general Japanese populations. In 1998–2000, rice samples were collected in 63 cities all over Japan and analyzed for Cd. The grand geometric mean for Cd in polished raw (uncooked) rice was 0.05 mg/kg and that rice accounts for approximately 40% of total dietary Cd intake (Shimbo et al. 2001). Phuong et al. (1999) collected various rice samples in Vietnam and some other parts of the country as well as from Yanco, Australia; the mean Cd concentrations of 0.008 and 0.011 mg/kg found for the Vietnamese rice samples and the analyzed Australian samples were also very low in Cd (< 0.003 mg/kg). In Swedish, a survey of the levels of Cd in different types of rice available on the Swedish retail market was carried out in 2001-2003. The types of rice included long and short grain, brown, white, and parboiled white rice; the mean levels of Cd were 0.024 mg/kg (Jorhem et al. 2008).In China, the Cd levels are slightly higher than Taiwan; a survey of the levels of Cd in milled rice sold on the Chinese market was carried out from 2005 to 2008 by Qian et al. (2010). The mean Cd level is 0.05 mg/kg (range < 0.001–0.74 mg/kg) and 97.8% of total samples were acceptable on Cd contamination level (0.2 mg/kg). Of note, samples from contaminated sites in China revealed elevated Cd levels in rice (range 0.2–2.4 mg/kg) (Nordberg 2003; Wang et al. 2001; Yanai et al. 1998; Yang et al. 2006).

In these studies conducted outside of Taiwan, the Cd levels are significantly lower, except for China. The reason for Taiwan’s higher Cd value could be due to industrial pollution in the sampling sites. However, Yilan, one of the sampling sites, is located on Lanyang Plain which has mainly developed agriculture and agricultural business activities. So the higher Cd could not possibly be caused by industrial pollution. A number of soil factors have been shown to govern the uptake of Cd by plants; of these, pH is probably the most important. The Cd adsorptive capacity of soils approximately triples for each unit increase in pH over the interval of pH 4–7 (Christensen 1984; Grant et al. 1999). We found the soils in Yalin have a slightly acidic pH of 4.5–5.0 (https://farmcloud.tari.gov.tw/SOA/index.aspx); this may increase the solubility of Cd and increase Cd concentration in crops. Nevertheless, pH alone cannot help explain why Cd in rice showed an increasing trend from 2010 to 2018. We will need to conduct further investigation to clarify.

Although our current results from Taiwan show that the Cd levels in rice do not exceed the maximum permitted value of 0.4 mg/kg, this still creates the potential for continuous exposure to Cd over a lifetime especially since rice is the staple food in Taiwan. Hence, Cd contamination should continue to receive attention.

### Estimate of Cd intake

Table 2 presents the consumers-only consumption rate of rice according to age in Taiwan. Data on consumption rates were obtained for males and females grouped into seven age groups: 0–3 years, 4–6 years, 7–12 years, 13–15 years, 16–18 years, 19–65 years, and > 65 years. Values for body weight were also derived through extrapolations of the data obtained from the NAHSIT. The distribution of Cd concentration and the LADD and HI were estimated by Monte Carlo simulation. For the consumer-only male group (age 19–65 years) alone, the P50 LADD of Cd was 0.06 μg/kg bw per day, and the HI P50 and P95 were 0.16 and 1.54, respectively.

**Table 2:** Daily consumption rate of rice and its products according to age in Taiwan

Group	Consumer only		
	Daily consumption (g/day)	Consumption range (g/day)	Body weight (kg)
0–3 years, male	LN (83.6, 69.1)	1.3–382.0	LN (12.99, 3.11)
0–3 years, female	LN (69.9, 90.4)	3.3–376.8	LN (12.84, 3.18)
4–6 years, male	LN (110.0, 132.8)	10.5–692.5	LN (21.00, 4.55)
4–6 years, female	LN (81.1, 85.1)	1.7–358.8	LN (20.03, 4.42)
7–12 years, male	LN (115.8, 88.2)	7.4–741.8	LN (34.77, 10.97)
7–12 years, female	LN (83.9, 68.5)	3.4–440.3	LN (33.22, 10.66)
13–15 years, male	LN (148.5, 127.2)	2.3–751.6	LN (57.62, 15.12)
13–15 years, female	LN (96.5, 94.6)	6.2–475.5	LN (50.44, 10.60)
16–18 years, male	LN (162.0, 209.2)	1.0–1,428.3	LN (65.98, 15.10)
16–18 years, female	LN (94.8, 88.6)	3.7–845.0	LN (54.42, 11.06)
19–65 years, male	LN (171.2, 170.4)	4.4–1,923.0	LN (69.33, 11.07)
19–65 years, female	LN (108.4, 115.8)	2.1–725.2	LN (57.27, 9.75)
> 65 years, male	LN (182.1, 144.4)	12.9–859.1	LN (63.83, 9.90)
> 65 years, female	LN (142.9, 88.1)	4.1–1,004.3	LN (57.07, 9.68)

Data are presented as (mean, standard deviation)

*LN* log-normal distribution

Table 3 shows the estimated Cd intake through rice. In the P50, Cd intake from rice ranged from 0.04 μg/kg bw per day (females aged 16–18 years) to 0.16 μg/kg bw per day (males aged 0–3 years). In the P95 of consumers, Cd intake from rice ranged from 0.39 μg/kg bw per day (females aged 16–18 years) to 1.34 μg/kg bw per day (males aged 0–3 years); these values exceeded the 0.36 μg/kg bw per day recommended by the EFSA in 2011. Moreover, in the P95 of consumers, all HI values for exposure to Cd in adults and children were > 1.

**Table 3:** Estimated cadmium lifetime average daily dose (LADD; μg/kg bw/day) and hazard index (HI) for rice and its products

Group	Consumer only					
	LADD P50	LADD P90	LADD P95	HI-P50	HI-P90	HI-P95
0–3 years, male	0.16	0.65	1.34	0.44	1.82	3.79
0–3 years, female	0.11	0.54	1.23	0.30	1.49	3.33
4–6 years, male	0.11	0.53	1.20	0.31	1.47	3.30
4–6 years, female	0.09	0.39	0.84	0.25	1.11	2.35
7–12 years, male	0.09	0.35	0.74	0.24	1.00	2.07
7–12 years, female	0.06	0.27	0.57	0.18	0.75	1.59
13–15 years, male	0.06	0.27	0.56	0.18	0.74	1.55
13–15 years, female	0.04	0.19	0.41	0.11	0.50	1.12
16–18 years, male	0.05	0.25	0.60	0.13	0.70	1.66
16–18 years, female	0.04	0.18	0.39	0.11	0.50	1.08
19–65 years, male	0.06	0.25	0.55	0.16	0.69	1.54
19–65 years, female	0.04	0.19	0.41	0.11	0.52	1.17
> 65 years, male	0.07	0.28	0.57	0.20	0.78	1.61
> 65 years, female	0.07	0.25	0.49	0.19	0.69	1.38

*P50* 50th percentile, *P90* 90th percentile, *P95* 95th percentile

In other countries, Nawab et al. (2018) investigated the concentrations of Cd in the fruits, vegetables, and cereals collected from different markets of Khyber Pakhtunkhwa, Pakistan. Their results showed that the highest mean concentration was 0.27 mg/kg in vegetables followed by fruits and cereals. In addition, the total HI values (fruits, vegetables and cereals) for Cd for both adults and children were > 1 and may pose a potential risk for the community consuming these foodstuffs on a daily basis.

Yu et al. (2017) investigated the concentrations of Cd in foods via surveys and a literature review, and found Cd ranged between 0.006 and 0.07 mg/kg. The concentrations of Cd in different food groups were in the decreasing order of meat > aquatic products > cereal > vegetable > bean > egg > dairy > fruit. Among all food groups, cereal was the most significant contributor to the dietary intake of Cd, followed by vegetable, aquatic products, and meat. For Chinese consumers, the results of risk assessment for all groups by the deterministic method and the probabilistic method showed the mean weekly Cd intake via dietary exposure was lower than the provisional tolerable weekly intake (PTWI) proposed by WHO. However, the 95th and 97.5th percentile total HI values all exceeded 1.

Al-Saleh and Abduljabbar (2017) determined levels of Cd and their potential health risks to residents in 37 brands of imported rice commonly consumed in Saudi Arabia after soaking and rinsing with water. The mean levels of Cd in soaked rice grains were 0.015 mg/kg dry weight, and none of the soaked, rinsed, unsoaked or unrinsed grains exceeded the acceptable level of 0.1 mg/kg set by the European Commission (EC 2006).

Pastorelli et al. (2018) was investigated Cd level in the most consumed rice varieties (*Oryza sativa* L.) to assess the health risk related to consumption of the cereal for Italian population. Cd concentrations were found to be below the maximum levels (MLs) established by the European Union level for rice (0.092 mg/kg for brown rice and 0.062 mg/kg for white rice). This result indicated that Cd exposure through the consumption of Italian rice does not represent a problem for the Italian population.

In populations in the European Union, several reports show that Cd level from rice consumption contributes to less than 10% of the tolerable weekly intake recommended by EFSA (Jorhem et al. 2008; Martorell et al. 2011; Perelló et al. 2015). Praveena and Omar (2017) investigated the levels of both trace elements and heavy metals in rice in Malaysia. For the non-carcinogenic health risk, the HI of exposure to Cd in adults and children were < 1.

In the present research, we only focused on locally grown rice and did not assess other types of products; we will consider to estimate Cd exposure from other foodstuffs in the future.

In present study, a beta binomial normal model was applied to the probabilistic assessment. Various scenarios for possible MLs in rice were selected to assess the impact of different MLs on Cd concentration and intake. More than 70% of children aged 2–6 years and > 30% of the general population have a daily dietary intake of Cd above the provisional tolerable daily intake.

The high LADD and HI values in the P95 group could be ascribed to the greater amount of food consumed. However, doses higher than the RfD are associated with a higher probability of producing an adverse effect. This warrants further investigation owing to the higher intake of Cd. Further investigations on Cd intake through other foods common in the Taiwanese diet (e.g., fruits and vegetables) may identify other issues, especially taking into consideration the vulnerability of infants. The distribution of Cd concentration and the LADD and HI were estimated by Monte Carlo simulation.

The results of this study show that consumers in the general population may not be subjected to the adverse health effects of exposure to Cd via the consumption of rice. However, the intake of Cd from other grains should also be evaluated in the future to provide a more comprehensive risk assessment considering all foodstuffs. According to the current data, the intake of Cd from rice may cause health problems in the high-end percentile (P95) group of consumers. The HI of the P95 for each age group was > 1, despite the conservative scenario assumptions.

Soaking or rinsing grains before consumption have been reported to reduce the levels of heavy metals and can minimize the non-carcinogenic health risks to residents from Cd (Al-Saleh and Abduljabbar 2017). We suggest consumers implement soaking or rinsing grains before consumption to reduce the levels of Cd and lower the non-carcinogenic health risks.

Taiwan’s regulation for Cd in rice is the same as the Codex Alimentarius Commission (2006) standard for Cd in rice (0.4 mg/kg); this value is double the standard established in China (0.2 mg/kg). When compared with countries outside of Asia, the Taiwanese population tends to consume markedly greater quantities of rice than those recorded in other parts of the world. As a result, we suggest Taiwanese government to adopt the standard used in China, since lowering the Cd MLs may have a strong impact on the intake of the contaminant from rice by Taiwanese consumers.

**Fig. 3:**
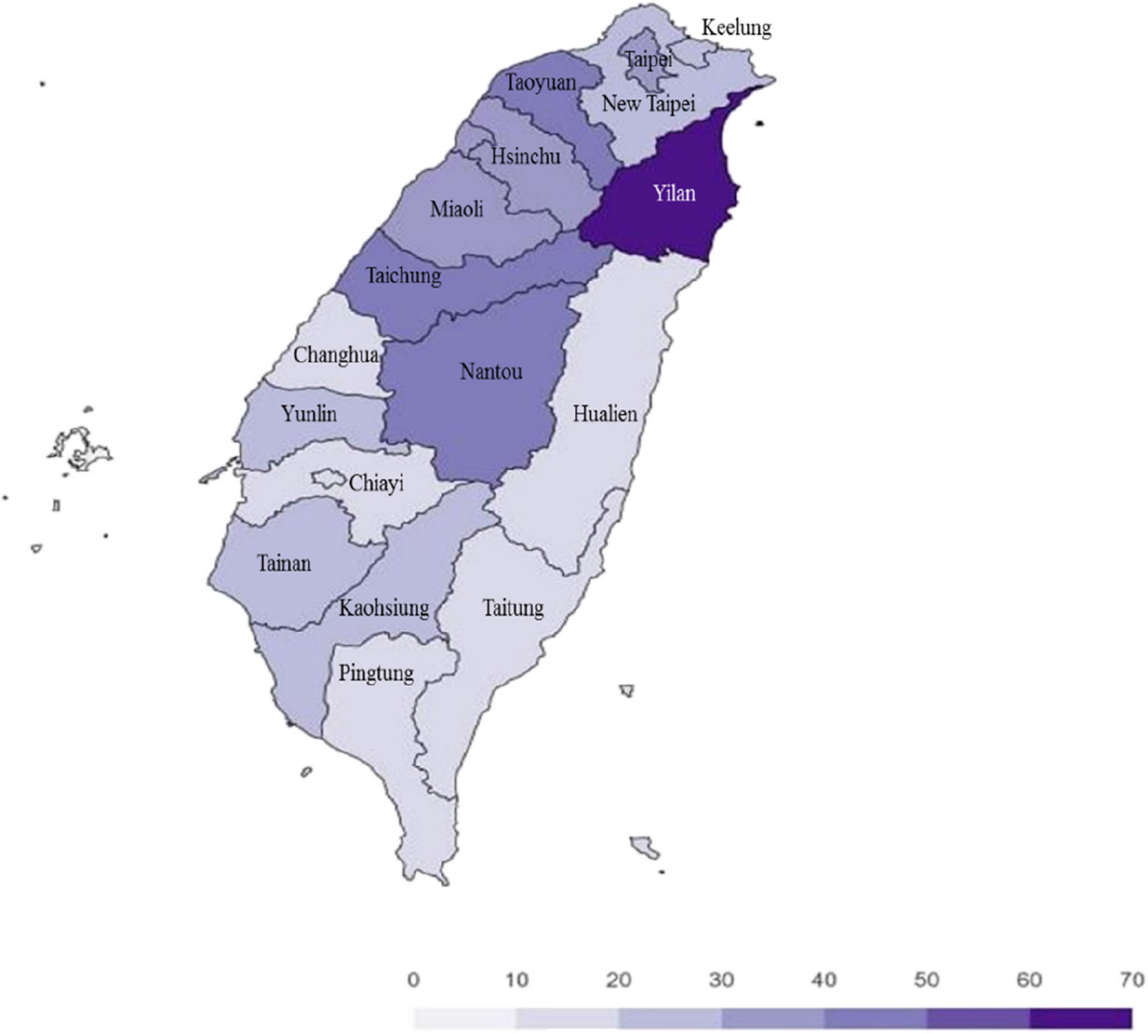
Hazard index (%) heatmap for cadmium consumed through rice in Taiwan: 19–65-year-old consumers only (50th percentile)

According to the HI heatmap for the daily intake of Cd from rice in Taiwan (Fig. 3), in the P50 of consumers, the HI for the various region were 0.19 (Changhua), 0.17 (Chiayi), 0.38 (Hsinchu), 0.18 (Hualien), 0.21 (Kaohsiung),0.28 (Keelung), 0.32 (Miaoli), 0.42 (Nantou), 0.23 (New Taipei City), 0.16 (Pingtung), 0.40 (Taichung), 0.20 (Tainan), 0.32 (Taipei), 0.14 (Taitung), 0.47 (Taoyuan), 0.63 (Yilan), and 0.21 (Yunlin). The highest risk of Cd from rice was observed in the Yilan area, whereas the lowest was noted in the Taitung area.

### Limitations

The present study has some limitations and research uncertainties. First, we did not follow a planned sampling approach, but randomly sampled rice from local farmers’ associations or markets; although we were certain that the rice was produced in Taiwan. However, the rice samples were not representative of all brands consumed in these regions of Taiwan. The content of heavy metal Cd was only measured in raw rice without soaking or rinsing the rice prior to cooking. The Cd levels may be reduced after soaking, rinsing, or other treatments.

Second, we did not assess other types of products, such as processed rice products, seafoods, vegetables, fruits, and other foods that may also be contaminated with Cd. Furthermore, we did not examine the risk in imported rice. Additional studies are warranted to establish whether the HI for Cd will remain < 1 in the general population, following the inclusion of other foods and beverages in the calculation of risk. The HI did not account for interactions with other metals; hence, the health hazard may be either under- or over-estimated. Comparison of our data with those of other studies was limited due to the differences in sample preparation, collection, and rice consumption.

Finally, although rice is the staple food of Taiwan, the consumption data from the 24-h dietary recall and food frequency questionnaires may be subject to memory information bias. In addition, the risk was assessed using rice intake and body weight data from the National Food Consumption Database (NAHSIT 2018) rather than from the local population. Future studies could analyze these data and develop a monitoring plan for evaluating the health risk associated with Cd in different foodstuffs in Taiwan.

## Conclusions

Our results indicate that, in the general population, the current levels of Cd in rice consumed in the Taiwanese diet do not constitute a public health and safety concern. However, further investigations are warranted to establish whether the HI for Cd will remain < 1 in the general population following the inclusion of other foods in the calculation of risk. In the P95 consumers, the daily intake of Cd from rice was higher than the RfD (0.36 μg/kg bw per day). This may present a risk for adult and children consumers. Long-term consumption of rice contaminated with Cd can pose potential health risks to the Taiwanese population, especially vulnerable groups (i.e., pregnant women, children, older adults, and patients). We recommend continuous monitoring of the Cd contamination levels in rice and the implementation of preventive measures.

According to the HI heatmap for the exposure of the general population in Taiwan to Cd from rice, the highest fraction of Cd was noted in the Yilan area (HI 0.64). Therefore, rice production in the Yilan area should be further monitored to evaluate the levels of Cd contamination. We could also lower the Cd MLs to the standard established in China. This approach may lower the intake of Cd from rice by Taiwanese consumers.

## Data Availability

The data that support the findings of this study are openly available in Taiwan Food and Drug Administration at https://data.fda.gov.tw/.
